# Making the invisible visible: the availability and desirability of adherence data in routine CF care– findings from a national questionnaire survey

**DOI:** 10.12688/f1000research.21033.2

**Published:** 2020-01-17

**Authors:** Louisa Robinson, Chin Maguire, Zhe Hui Hoo, Martin J. Wildman

**Affiliations:** 1 Clinical Trials Research Unit, University of Sheffield, Sheffield, South Yorkshire, S1 4DA, UK; 2 Sheffield Adult CF Centre, Sheffield Teaching Hospitals NHS Foundation Trust, Sheffield, Yorkshire, S5 7AU, UK; 3 School of Health and Related Research, University of Sheffield, Sheffield, Yorkshire, S1 4DA, UK

**Keywords:** cystic fibrosis, medication adherence, routine monitoring, nebulisers

## Abstract

**Background:** Inhaled medications for cystic fibrosis (CF) are effective but adherence is low. Clinicians find it difficult to estimate how much treatment people with CF (PWCF) take, whilst objective adherence measurement demonstrates that patients are poorly calibrated with a tendency to over-estimate actual adherence. The diagnostic approach to a PWCF with deteriorating clinical status and very low adherence is likely to be different to the approach to a deteriorating patient with optimal adherence. Access to objective adherence data in routine consultations could help to overcome diagnostic challenges for clinicians and people with CF. Attitudes of clinicians to the use and importance of routinely available adherence data is unknown.

**Methods:** We conducted an online questionnaire survey with UK CF centres. We asked five questions relating to the current use and perception of objective measurements of adherence in routine care.

**Results:** A total of eight CF centres completed the questionnaire. Few of the responding centres have adherence data readily available in routine clinics (13% of centres use medicines possession ratio; of centres with access to I-nebs® it was estimated that 17% of patients had I-neb data regularly available in clinics). All centres considered the availability of objectively measured adherence data to be important. Respondents identified that systems developed to provide adherence data in clinical practice must provide data to both clinicians and patients that is readily understood and easy to use.

**Conclusions:** Centres perceived the availability of adherence data in routine care to be important but objective measures of adherence is rarely available at present.

## Introduction

Cystic Fibrosis (CF) is a multi-system life-limiting genetic condition in which the most common cause of death is respiratory failure. Daily use of inhaled mucolytic and antibiotic medications are effective to prevent pulmonary exacerbations and preserve lung function ^1, 2^. Low adherence to CF medications is associated with poorer health outcomes ^3^, yet real-world adherence to inhaled medications is only 35–50% ^4, 5^. 

Another problem is the potential for diagnostic uncertainties – in a study where the objectively measured adherence was 36%, people with CF reported median adherence of 80% whilst clinicians estimated adherence rate of 55–60% ^5^. This disparity makes it challenging for healthcare professionals to make informed clinical decisions. The ‘invisibility’ of adherence in routine care can result in consultations characterised by the ‘lamp post syndrome’ ^6^, whereby there is a tendency for clinicians to use readily available information which can be misleading. In other words, clinicians ended up seeking information “where the light is” rather than to use all relevant data sources. Since neither patient self-report nor clinician estimation provides an accurate indication of medication adherence, adherence will be invisible in those centres without systems that make objectively measured adherence visible. This lack of adherence data is critical for diagnosis and assessment. Indeed, the approach in a patient with deteriorating clinical status and low adherence (where treatment failure is due to non-use of existing treatments) is very different to the approach in a patient with deteriorating clinical status and high adherence (where existing treatments have failed, and treatment escalation must be considered).

Furthermore, enabling self-monitoring using objective feedback is associated with increases in target health behaviours ^7^ and has been identified as a facilitator in nebuliser adherence ^8^. Access to, and feedback from, accurate adherence data could facilitate more effective self-monitoring and self-care for people with CF.

Despite evidence pointing to the importance of objectively measured adherence data in routine CF care, it remains unclear how frequently clinicians readily have access to objective adherence data in their day-to-day practice and whether such access is desired by clinicians. We therefore conducted an online survey among UK CF centres to establish the perceived importance of routinely available adherence data, and the extent to which this data is currently used in routine care.

## Methods

Centre directors from all 29 UK adult CF centres were invited by email on 12 March 2019 to participate in a short online questionnaire about their views and practices in using adherence data in their day-to-day management of people with CF. Where available, contact details were sought through the CF registry; in the absence of this, email addresses were ascertained through hospital websites.

Centre directors were encouraged to discuss the questions with their clinical colleagues before responding. Centres that did not respond after two weeks were sent two reminders, at two week intervals. All responses were collected within two weeks of the final reminder. 

The participant information sheet detailed that responses to the survey would be used for research purposes. The information sheet was embedded within the invitation email. Consent to participate was implied through completion of the questionnaire. The questionnaire was designed with Qualtrics© 2019 software (version March 2019, Provo, Utah) and consisted of five-items with a mixture of Likert scale, percentage estimate and free-text responses (see *Extended data*). Respondent Internet Protocol (IP) addresses were captured and used to check for duplicate responses. No duplicate responses were captured.

Responses to Likert scale questions (Q1, Q4) were coded 1–5 (1= Very important, 5= Not important) and medians and inter-quartile ranges summarised. For responses to questions requesting a percentage estimate (Q2, Q3), means and standard deviations were calculated. Free-text responses (Q5) were summarised by extracting key themes.

The study received approval from the School of Health and Related Research (ScHARR) Research Ethics Committee, University of Sheffield (ref: 024042). The survey was hosted on Qualtrics (survey tool approved by the University of Sheffield Corporate Information and Computing Services). The University of Sheffield was the data controller and all survey data exported for analysis was stored on an access restricted folder on the University shared file store.

## Results

A total of eight adult UK CF centres (28%) provided data from sites across England, Northern Ireland and Wales. Summaries by questionnaire item are summarised in Table 1.

**Table 1.** Questionnaire responses summarised by item (excluding free-text responses to question 5).

Question	n	Median (IQR)	n responses (%)
Q1: A clinical vignette to ascertain whether clinicians desire objective adherence data at the point of consultation	7	2 (1 – 2)	Very important: 2 (29%) Important: 4 (57%) Moderately important: 1 (14%)
Q4: The perceived importance of a system which provides objective adherence data at the point of consultation How important is it to have such a system in your centre?	6	1.5 (1 – 2)	Very important: 3 (50%) Important: 3 (50%)
*1= “Very important”, 2= “Important”, 3= “Moderately important”, 4= “Slightly important”, 5= “Not important”*			
	n	Mean (SD)	
Q2: Proportion of adults whereby pharmacy refill data is readily available at the point of consultation	6	13% (31%)	
Q3: Proportion of adults whereby I-neb® data is readily available at the point of consultation	5	17% (21%)	

An average of 87% of patients using inhaled therapies were estimated not to have up to date medicines possession ratio or pharmacy refill data available during a typical outpatient clinic. This included four centres that said 100% of their patients would not have this information available. Across five centres, an average of 83% of patients were estimated not to have I-neb® data readily available at the point of consultation. It was stated by two sites that I-nebs were not used at their centre and it is important to recognise that at the time the study, the only routinely available devices to collect objective adherence data were Inebs®.

In contrast to low availability of objective adherence data, all centres considered having up-to-date, objective adherence data for inhaled therapies at the point of diagnosis to be at least moderately important; 57% considered this to be important and a further 29% considered this to be very important. When asked if they thought that a system able to automatically collect and provide objective, up-to-date adherence data within a consultation would be important, all responding centres said that this would be important to have in their centre and 50% of respondents felt this would be very important.

A total of seven free-text responses were received for question five regarding the key features for a system to routinely collect objective adherence data. Multiple respondents stated that “ease of use” would be important, as well as quick and reliable data access. Centres also stated that it would be important for data to be available for both patients and staff to facilitate discussions on adherence.

## Conclusion

Adherence is important for accurate diagnosis, treatment planning and self-management in people with CF ^3, 6, 8, 9^. We have demonstrated that whilst centres providing care to people with CF perceive objective adherence data to be important, comprehensive provision of these data is not a feature of current CF clinical practice. Only a small percentage of people with CF have objective measures of adherence available for consultations (between 13–17% depending on the use of medication possession ratio or I-neb®), with this figure likely to be even lower among centres which did not respond to the survey. This is despite an overall consensus that access to objective adherence data is important for making accurate diagnoses. If a system could produce up-to-date, routinely collected objective adherence data for use in consultations, all centres which responded felt this would be an important asset.

The results of this study demonstrate that in the responding centres clinicians felt that objectively measured adherence at the point of consultation would be useful but was rarely available. The situation in CF contrasts with that of other long-term respiratory conditions such as asthma whereby adherence measurement is mandated prior to commencing expensive biologics e.g. mepolizumab ^10^. To help embed an objective adherence monitoring system within routine CF clinical practice, it would be useful to ascertain the barriers for implementing such a system among CF centres in future surveys.

A limitation of the current study is that with only 8 out of 29 UK adult centres responding. Centres with less interest in monitoring adherence may be less likely to respond, so it is uncertain whether objective adherence data is considered important across the whole of the UK. Nevertheless, it is notable that within the centres that did respond, there was a consensus that objective adherence data was important and no centres that considered objective adherence data to be unimportant.

## Ethical approval

Ethical approval for the collection and analysis of data was obtained from School of Health and Related Research (ScHARR) Research Ethics Committee, University of Sheffield (reference: 024042)

## Data availability

### Underlying data

ORDA: Findings from a national questionnaire survey of UK CF centres on availability and desirability of routinely available adherence data, https://doi.org/10.15131/shef.data.10059251.v1 ^11^.

This project contains the following underlying data:

- CSV file exhibiting the responses to the questionnaire for the eight centres

### Extended data

ORDA: Findings from a national questionnaire survey of UK CF centres on availability and desirability of routinely available adherence data, https://doi.org/10.15131/shef.data.10059251.v1 ^11^.

This project contains the following extended data:

- Questionnaire sent to the CF centers

Data are available under the terms of the Creative Commons Zero “No rights reserved” data waiver (CC0 1.0 Public domain dedication).
