# Supplementary material for: Making the invisible visible: the availability and desirability of adherence data in routine CF care– findings from a national questionnaire survey
Source: F1000Res. 2020 Jan 17;8:1904. Originally published 2019 Nov 11. [Version 2] doi: 10.12688/f1000research.21033.2 (PMC6996524; doi:10.12688/f1000research.21033.2)
Supplement: Peer review history

# Peer review: Reviewer response for version 2

**DOI:** 10.5256/f1000research.24331.r58711

**Published:** 2020-01-31

**Authors:** Mark Hew

**Affiliations:** 1 Alfred Health, Melbourne, Vic, Australia; 2 School of Public Health and Preventive Medicine, Monash University, Monash, Australia

Comments satisfactorily addressed.

I confirm that I have read this submission and believe that I have an appropriate level of expertise to confirm that it is of an acceptable scientific standard.

# Peer review: Reviewer response for version 1

**DOI:** 10.5256/f1000research.23149.r58100

**Published:** 2020-01-03

**Authors:** Mark Hew

**Affiliations:** 1 Alfred Health, Melbourne, Vic, Australia; 2 School of Public Health and Preventive Medicine, Monash University, Monash, Australia

This manuscript describes a survey of adherence monitoring availability, and views regarding the benefits of such, in the context of UK national CF centres.

 Aims, methods, results are described in sufficient detail for the reader to understand and follow. The discussion is reasonable and does not exceed the scope of the data.

 The results demonstrate substantial limitations in clinicians’ ability to capture and utilise adherence data in clinical care- findings important to highlight and necessary to address.

 I have the following major comments:

1. The 8/29 response rate from centre directors is disappointing. This may therefore represent significant selection bias and this bias should be explicitly mentioned in the limitations; i.e. centres with less interest in an adherence monitoring system are less likely to respond.
2. The sub-optimal response rate may also indicate limited interest or engagement among some centres for implementing a concerted adherence monitoring system. The authors might consider including this in their discussion, in a sensitive and tactful manner.
3. While the respondents agreed a user-friendly system to make adherence data available in consultations was desirable, they were not asked what barriers currently stood in the way of implementing this- (staff, equipment, time). If this survey is repeated in future, this would yield useful information to develop and implement such a system.
4. As a practitioner outside the CF field, I am surprised that adherence monitoring is not a routine part of routine assessment, especially prior to the use of eye-wateringly expensive advanced therapies. For example, the Severe Asthma centres in the UK are tasked with ensuring objective adherence data is acceptable prior to commencing biologics (far less expensive than gene potentiators), and the Australian Pharmaceutical benefits system requests that adherence is addressed prior to prescription of biologics (although objective measurement is not a requirement here). While clinicians are naturally (and, I judge, correctly) reluctant to set adherence thresholds requirements prior to implementing advanced therapies, knowledge of adherence patterns is always desirable when contemplating stepping-up any therapy. The authors may consider drawing comparisons between the practice in CF and severe asthma fields even in the UK alone.

I confirm that I have read this submission and believe that I have an appropriate level of expertise to confirm that it is of an acceptable scientific standard, however I have significant reservations, as outlined above.

# Response

**Published:** 2020-01-17

**Authors:** Louisa Robinson

**Affiliations:** CTRU, ScHARR, The University of Sheffield, UK

Dear Professor Hew,

Please see our responses to your comments below and refer to the revised article which has now been published.

1. The 8/29 response rate from centre directors is disappointing. This may therefore represent significant selection bias and this bias should be explicitly mentioned in the limitations; i.e. centres with less interest in an adherence monitoring system are less likely to respond.

**We agree this is an important bias and have now included this in the new 3rd paragraph of the conclusion. The sentence now reads:**   

**"Centres with less interest in monitoring adherence may be less likely to respond, so it is uncertain whether objective adherence data is considered important across the whole of the UK."**

2. The sub-optimal response rate may also indicate limited interest or engagement among some centres for implementing a concerted adherence monitoring system. The authors might consider including this in their discussion, in a sensitive and tactful manner.

**We agree this is an important point and have now added this in the 1st paragraph of the conclusion.** **The sentence now reads:**

**"Only a small percentage of people with CF have objective measures of adherence available for consultations (between 13–17% depending on the use of medication possession ratio or I-neb®) with this figure likely to be even lower among centres which did not respond to the survey."**

3. While the respondents agreed a user-friendly system to make adherence data available in consultations was desirable, they were not asked what barriers currently stood in the way of implementing this- (staff, equipment, time). If this survey is repeated in future, this would yield useful information to develop and implement such a system.

**This is a good suggestion and we have now added the following sentence in the 2nd paragraph of the conclusion** **:**

**"To help embed an objective adherence monitoring system within routine CF clinical practice, it would be useful to ascertain the barriers for implementing such a system among CF centres in future surveys."**

4. As a practitioner outside the CF field, I am surprised that adherence monitoring is not a routine part of routine assessment, especially prior to the use of eye-wateringly expensive advanced therapies. For example, the Severe Asthma centres in the UK are tasked with ensuring objective adherence data is acceptable prior to commencing biologics (far less expensive than gene potentiators), and the Australian Pharmaceutical benefits system requests that adherence is addressed prior to prescription of biologics (although objective measurement is not a requirement here). While clinicians are naturally (and, I judge, correctly) reluctant to set adherence thresholds requirements prior to implementing advanced therapies, knowledge of adherence patterns is always desirable when contemplating stepping-up any therapy. The authors may consider drawing comparisons between the practice in CF and severe asthma fields even in the UK alone.

**This is a very important point and we have now drawn such a comparison in the 2nd paragraph of the conclusion** **:**

**"The situation in CF contrasts with that of other long-term respiratory conditions such as asthma whereby adherence measurement is mandated prior to commencing expensive biologics e.g. mepolizumab."**

# Peer review: Reviewer response for version 1

**DOI:** 10.5256/f1000research.23149.r57625

**Published:** 2019-12-30

**Authors:** Dominique Pougheon Bertrand

**Affiliations:** 1 LEPS EA 3412, University of Paris 13, Bobigny, France

This article deals with the adherence of adult patients with CF to the nebulization treatments and more particularly the importance of assessing their adherence in clinic consultations to establish a good diagnosis of the patient’s health (a deterioration might be due to non-adherence to a prescribed treatment or other causes despite good adherence to the treatments) and thus to offer the best treatment strategies to the patient.

 The purpose of the study is to assess the availability and desirability, for the clinicians involved in CF care in the UK, of objective adherence data from their patients. Five questions were asked through an online questionnaire survey to the UK Adult CF centres. Availability refers to Medical Possession Ratio (MPR - refill data) or I-neb data (a device used to nebulize various treatments) available during the clinic visit. Desirability refers to the value that clinicians place on this data at the point of consultation. 

 Though clinicians place importance on objective adherence data during the clinic visits of the patients, this data is rarely available (only available for about 15% patients) and their provision not a feature of CF care in current clinical practice. The author indicates as a limitation of the study the fact that the response rate to the survey is only 28% of the CF centers (8 centers out of 29).

 This paper is very clear, the methods is presented in details so it could be replicated in another study, and the results are fully presented.  It gives perspectives to improve both the quality of care and the health outcomes of the patients by increasing the availability and use of patient adherence data to nebulized treatments. This would increase the relevance of prescriptions and possibly open up a discussion with the patient about the feasibility of their treatments and routines necessary to sustain care in daily life.

 May the author consider citing the following paper (6 ^th^ position in the reference) as it is an assessment of adherence in CF patients for inhaled therapies in France: Heloïse Rouzé *et al.* ^1^

I confirm that I have read this submission and believe that I have an appropriate level of expertise to confirm that it is of an acceptable scientific standard.

## References

[1] : Adherence to long-term therapies in cystic fibrosis: a French cross-sectional study linking prescribing, dispensing, and hospitalization data. *Patient Prefer Adherence*.2019;13: 10.2147/PPA.S211769 1497-1510 10.2147/PPA.S211769 31564837PMC6732572
